# Earliest Directly-Dated Human Skull-Cups

**DOI:** 10.1371/journal.pone.0017026

**Published:** 2011-02-16

**Authors:** Silvia M. Bello, Simon A. Parfitt, Chris B. Stringer

**Affiliations:** 1 Department of Palaeontology, The Natural History Museum, London, United Kingdom; 2 Institute of Archaeology, University College London, London, United Kingdom; University of Oxford, United Kingdom

## Abstract

**Background:**

The use of human braincases as drinking cups and containers has extensive historic and ethnographic documentation, but archaeological examples are extremely rare. In the Upper Palaeolithic of western Europe, cut-marked and broken human bones are widespread in the Magdalenian (∼15 to 12,000 years BP) and skull-cup preparation is an element of this tradition.

**Principal Findings:**

Here we describe the post-mortem processing of human heads at the Upper Palaeolithic site of Gough's Cave (Somerset, England) and identify a range of modifications associated with the production of skull-cups. New analyses of human remains from Gough's Cave demonstrate the skilled post-mortem manipulation of human bodies. Results of the research suggest the processing of cadavers for the consumption of body tissues (bone marrow), accompanied by meticulous shaping of cranial vaults. The distribution of cut-marks and percussion features indicates that the skulls were scrupulously 'cleaned' of any soft tissues, and subsequently modified by controlled removal of the facial region and breakage of the cranial base along a sub-horizontal plane. The vaults were also ‘retouched’, possibly to make the broken edges more regular. This manipulation suggests the shaping of skulls to produce skull-cups.

**Conclusions:**

Three skull-cups have been identified amongst the human bones from Gough's Cave. New ultrafiltered radiocarbon determinations provide direct dates of about 14,700 cal BP, making these the oldest directly dated skull-cups and the only examples known from the British Isles.

## Introduction

The act of collecting and modifying human body parts after the death of an individual for trophy or ritual use is well documented [1]–[3]. Although examples of post-cranial modification are known, the majority of modified human remains are cranial elements [4]. In these examples, skinning of the skull and removal of brain and facial tissues has been attributed to cannibalism, trophy display or secondary burial [5]. The use of skull vaults as drinking cups is known from recent ethnographic studies as well as historical accounts [6]. Herodotus in *The Histories* (5^th^ century BC) portrayed the Scythians as people who drank from the skulls of their enemies. Similar traditions were described for China in *The Record of Great Historian* by Sima Qian (1^st^–2^nd^ centuries BC) and for Viking tribes by Mágnus Ólafsson in the *Krakumal* written in 1636. Human skull-bowls, known as *kapala* in Sanskrit, were fashioned from the oval upper section of a human cranium and used as a libation vessel for a number of Vajrayana deities in tantric Buddhist rituals [7]. Laufer [7] documented Historic evidence of the use of human calvariae as drinking bowls in India, where the ritual seems to be still practiced by the Aghori sub-sect (8; http://www.lightstalkers.org/images/show/137862). Skull-cups have been reported as being used by Australian aborigines [9]–[10], and in the nineteenth century human skulls were used as drinking cups in Fiji [11] and other islands in Oceania [4].

Despite this widespread geographical and temporal occurrence, archaeological evidence of skull-cup preparation is rare. Among the human remains excavated at the site of Nawinpukio in Peru (400–700 AD), one skull exhibits modifications, such as percussion marks, cut-marks and a polished border, which are consistent with its use as a bowl [12]. Earlier skull-cups have been described from Bronze Age sites in Europe. At Grotte du Pradel, a human cranial vault was found on the contemporary ground surface, surrounded by five burials [4]. Bronze Age modified cranial vaults from El Mirador Cave (Sierra de Atapuerca, Spain) are also consistent with skull-cups, although the authors excluded any symbolic behavior associated with the processing and consumption of the bodies [13]. Evidence from the Neolithic site of Herxhein in Germany [14]–[16] suggests that human cranial bones were systematically modified to produce skull-cups. So far, the earliest known evidence for skull-cup preparation is from the Upper Palaeolithic Magdalenian culture (∼15–12,000 yr BP, 17) in Europe. Nine cranial remains from the site of Le Placard Cave (Charente, France) have been interpreted as skull-cups. All show signs of defleshing, breakage by percussion, and careful ‘retouching’ of the broken borders [18]–[19]. Human remains from Isturitz (Gironde, France) are dominated by cranial elements, most of which have been cut-marked and some modified by percussion to make skull-cups [20]. Although several of these studies give detailed descriptions of how the skulls were modified, none clearly identify the sequence of manipulations involved in the manufacture of skull-cups. To our knowledge, none of these Magdalenian human skull-cup has been directly dated.

**Figure 1:**
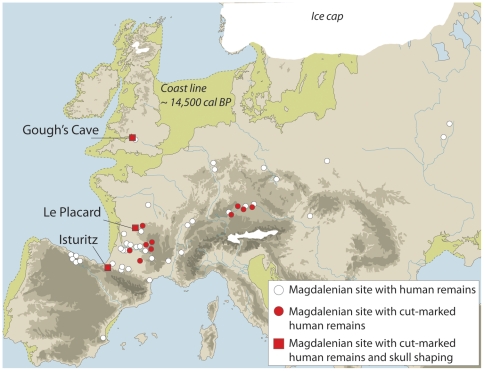
Palaeogeographical context of Gough's Cave and other Magdalenian sites with human bone remains.

At the Magdalenian site of Gough's Cave (Somerset, England; Figure 1), human and non-human remains were found discarded in the same archaeological context, exhibiting cut-marks and humanly-induced breakage. In the case of human remains, the interpretation of these modifications has been controversial [21]–[27]. Both natural damage and cannibalism were suggested, but no previous study has recognized the significance of the breakage pattern observed on the skulls.

In this paper we describe the post-mortem processing of the head and identify a range of modifications associated with the production of human skull-cups. From this evidence it is possible to identify the sequence of activities undertaken to make skull-cups at Gough's Cave. New ultrafiltered radiocarbon determinations [28] provide direct dates of about 14,700 cal BP, making these the oldest directly dated skull-cups.

## Results

The human cranial sample from Gough's Cave comprises 41 pieces (37 skull fragments and 4 mandible fragments). Most of these are small pieces of basicranium but the sample also includes substantial parts of the facial region, three almost complete mandibles and three cranial vaults (table 1, Supplementary Figures S1, S2, S3 and S4). Refitting was possible for 14 specimens (34.1% of cases), including re-fits between specimens found during different excavation campaigns.

**Table 1:** The Upper Palaeolithic cranial sample from Gough's Cave, Somerset.

Specimen	Year excavated			Left			Right			Left			Right			Left		Right	
		F	O	P	T	S	P	T	S	N	M	Z	N	M	Z	B	R	B	R
*Skull-cups*																			
GC 87 (190^a^, 162)	1987	•	•	•		•	•	•		•			•						
GC2 [1.1/4^b^, GC 87 (107, 90, 169^c^)]	1927–28, 1987	•	•	•			•	•	•										
GC3 [1.1/1]	1929	•	•	•	•	•	•												
*Neurocranial bones*																			
GC 86 (55) N, GC 87 (60) E	1986, 1987		•					•											
GC 87 (8, 55)	1987				•	•													
GC 86 (55) B	1986				•	•													
GC 87 (55) D	1987						•		•										
GC 87 (73)	1987							•	•										
GC 87 (138) B	1987							•	•										
GC 86 (55) A	1986		•																
GC 86 (un-numbered)	1986		•																
GC 86 (55) I	1986		•																
GC 86 (55) E	1986				•														
GC 86 (55) W	1986				•														
GC 7 (1.1/2)	1927–28						•												
GC 87 (16)	1987							•											
*Facial bones*																			
GC 87 (25, 29, 87)	1987										•	•		•					
GC 86 (55), GC 87 (139, 230B)	1986, 1987										•			•					
GC 87 (226)	1987													•	•				
GC 87 (141)	1987										•								
GC 87 (230) A	1987											•							
*Mandibles*																			
GC 87 (49)	1987															•	•	•	•
GC 6 (1.1/3)	1927–28															•	•	•	•
GC 87 (253)	1987															•		•	•
GC 86 (un-numbered) A, B	1986																•		

Dots indicate bones or parts of bones present (F, frontal; O, occipital; P, parietal; S, sphenoid; N, nasal; M, maxilla; Z, zygomatic; B, body; R, ramus). New radiocarbon determinations of two of the skull cups have used ultrafiltration to remove contaminants (28):

a OxA-17849. 12,590±50 BP,

b–c OxA-17848. 12, 485±50 BP.

Based on the analysis of cranial and dental remains, Humphrey and Stringer [29] suggest a minimum number of five individuals, while Hawkey [30] argues for a minimum number of seven individuals based on the lack of any clear association between the dental elements. For the purpose of this publication, a conservative approach was adopted and a minimum number of 5 individuals was counted: a young child (3.2 years old), two adolescents, an adult and an older adult [29].

The breakage pattern, light weathering and rarity of random striae on the human bones suggest that sediment pressure, weathering and trampling did not significantly alter the remains after their deposition [31]. Many of the pieces display incisions with micromorphological characteristics (e.g. internal microstriations, shoulder effect and hertzian cones)typical for cut-marks produced by a stone tool [32]–[34]. Most of the cut-marks are consistent with slicing, although some chopping marks are also present, but scrape marks are rare. Characteristic breakage features include percussion pits and striae, and lunate scars, some with adhering flakes and anvil striae, which are consistent with humanly-induced fractures on fresh (‘green’) bone [35]–[37].

Among the human cranial sample, the frequency of cut-marks was extremely high (95.1%), but less than a half (46.2%) of the cranial fragments showed percussion marks and associated fracture damage. On the skull, cut-marks were present only on the ectocranial surface. No fragments showed obvious burning damage. Modifications were observed on all individuals regardless of their age.

### Frontal bone

Three frontal bones were analysed, all of which had paracoronally/obliquely oriented slicing cut-marks on the squama [cf. 37]–[41]. Parasagittal cut-marks were present only on GC2 [cf. 14], [ 37]. The absence of muscular attachments on this area of the vault suggests that the cuts were produced during scalping.

The frontal bones also showed cut-marks along the temporal lines [cf. 19], [ 42]. These cut-marks were normally in sub-parallel groups, indicative of cutting of the *temporalis* muscle. In one case [skull GC87(190) + GC87(162)], percussion marks were observed between the left temporal line and the coronoid suture in the area separating the frontal bone from the great wing of the sphenoid.

**Figure 2:**
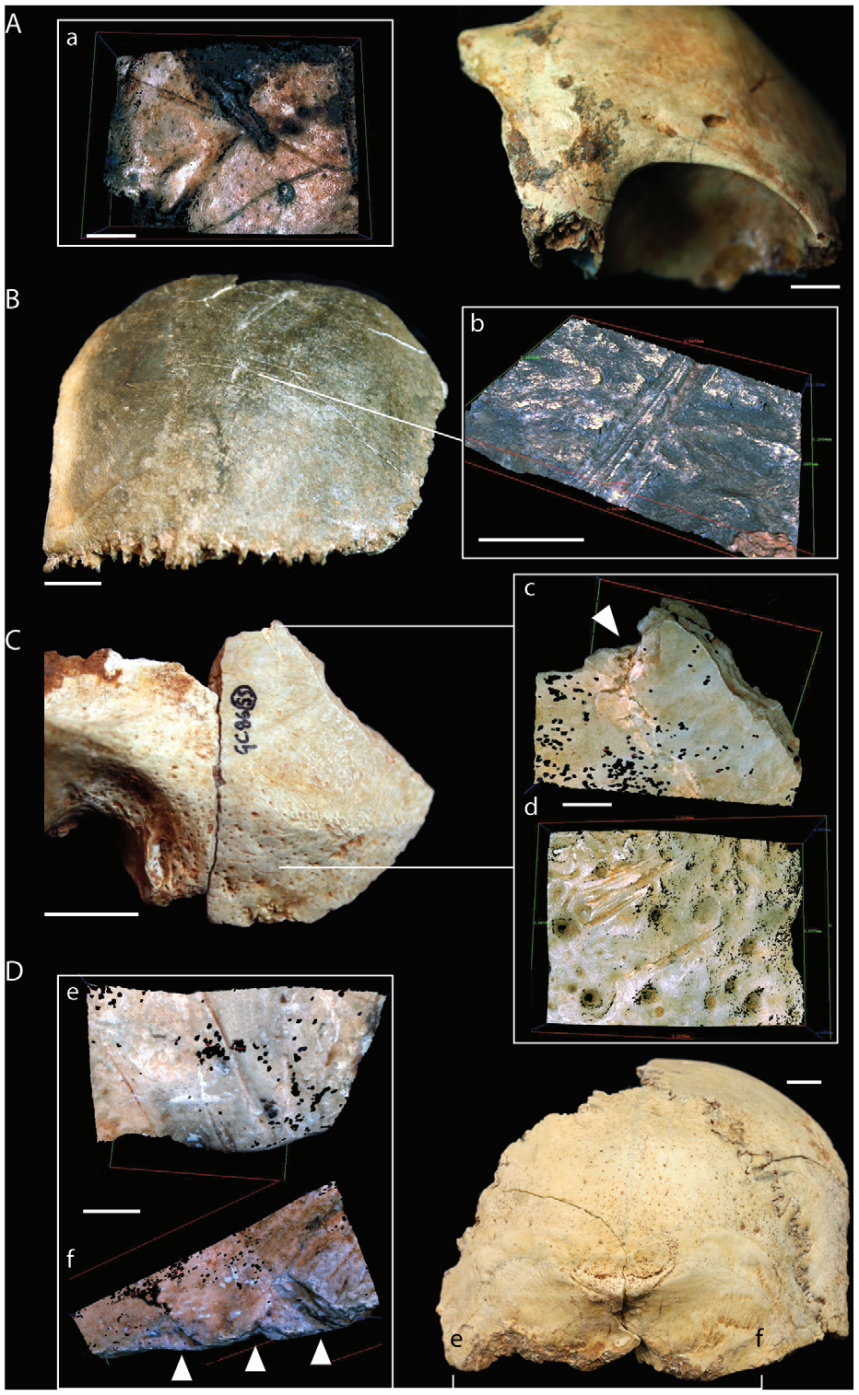
Cut-marks and percussion marks on cranial bones from Gough's Cave. (A) cut-marks on frontal bone (GC3); (B) cut-marks on parietal GC 7(1.1/2); (C) cut-marks and percussion marks on temporal bone [GC 87 (8, 55)]; (D) cut-marks and percussion marks on occipital bone (GC2) (scale  =  10 mm). (a–f), Alicona three-dimensional (3D) images of human modifications (scale  =  1 mm). Arrows indicate percussion marks.

**Figure 3:**
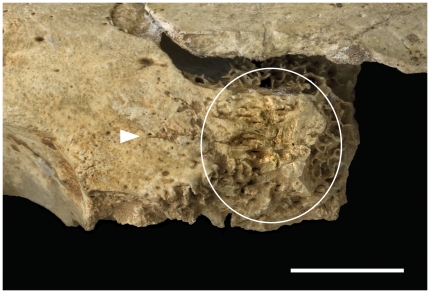
Cut-marks and percussion mark on a frontal bone from Gough's Cave. Cut-marks (white arrows) and percussion marks (circled) on the frontal bone of GC2 (scale  =  10 mm). The percussion damage (circled) is the result of a carefully placed blow along the fronto-nasal suture to separate the facial bones from the vault.

On two of the frontal bones, the area between the glabella and the nasion showed both paracoronally and parasagittally oriented cut-marks Figure 2A; [cf. 38]. In one case (CG2) cut-marks were associated with a series of vertical percussion marks resulting from multiple hits by a sharp stone Figure 3; [cf. 37]. The pattern of percussion damage suggests that the facial bones were separated from the vault by carefully placed blows along the fronto-nasal suture.

Clusters of cut-marks were observed on the supra-orbital margin and in the orbits [cf. 41]. Cut-marks inside the orbits, mainly radiating from the centre, are consistent with cutting of the *orbicularis oculi* muscle and associated extraction of the eye.

GC87(190) + GC87(162) showed sub-parallel abrasions on the ectocranial surface of the frontal bone, possibly resulting from contact between the skull and an anvil [cf. 37].

### Parietals

A total of 8 parietal bones (4 left and 4 right), were analysed. Groups of parasagittally oriented cut-marks of different lengths were found in the area of attachment of the *temporalis* muscle [cf. 14], [ 18]. Parasagittal cut-marks were also present along the sagittal suture in two cases [cf. 5]. Slicing cut-marks on the cranial vault were normally broad and quite shallow, with a cross-sectional profile indicating that the stone tool was held at an oblique angle to the bone surface (Figure 2B; 43). Their location and micro-morphological characteristics are indicative of scalping.

Three fragments also exhibit cut-marks on the posterior portion of the parietals, with an orientation parallel to the lambdoidal suture [cf. 39]. These indicate cutting of the insertion of the *sternocleidomastoid* muscle, during severing of the head.

**Figure 4:**
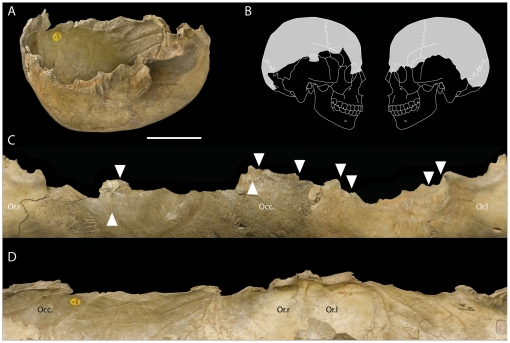
View of the modified edges of a skull-cup from Gough's Cave. (A) Skull-cup GC87(190 + GC87(162) (scale  =  50 mm) and (B) the portion of the cranial vault represented. (C) Exocranial and (D) endocranial views showing the modified edges of the vault. White arrows indicate percussion damage; Or.l  =  left orbit, Or.r  =  right orbit, Occ.  =  occipital.

Percussion marks were observed posterior to the coronoid suture, just above the temporo-parietal suture in two cases, and in one in proximity to the mastoid (postero-inferior) angle and the parietomastoid suture. This impact damage is associated with flaking and chipping of the edge, producing a series of curved projections (figure 4).

### Temporal

A total of 12 temporal bone fragments were analysed, nine of which had cut-marks (75%), and five (41.7%) had percussion marks and associated fracture damage (Figure 2C). The degree of fragmentation varied considerably, from almost complete, to small fragments of the petrous pyramid. None of the temporal bones has an intact zygomatic process.

Paracoronally oriented cut-marks were present on the squama of the temporal, probably produced during removal of the *temporalis* muscle. In six cases, short cut-marks were observed at a right angle to the root of the zygomatic process [cf. 37]. These were probably inflicted during the severing of the ears. Finally, in three cases, cut-marks were observed on the supramastoid crest (Figure 2C, d) in proximity to the occipitomastoid sutures [cf. 42]. Detachment of the head is indicated by cut-marks on the posterior areas of the parietals, particularly those associated with the insertion of the *sternocleidomastoid* muscle [cf. 44].

Crushing and associated abrasion at the root of the zygomatic process were present on one fragment, GC87[16]. Percussions marks were observed close to the parietal notch, anteriorly to the zygomatic process and on the petrous portion of the temporal along an almost sub-horizontal plane.

### Sphenoid

Nine fragments of sphenoid (6 left and 3 right) were analysed, six of which are cut-marked (66.7%). No percussion marks were observed.

Paracoronally oriented cut-marks in the area of the greater wing of the sphenoid indicate removal of the *temporalis* muscle. In three cases, sub-parallel slicing cut-marks were concentrated on the infratemporal crest of the greater wing and could relate to the cutting of the *medial pterygoid* muscle during detachment of the mandible from the skull [cf. 44].

### Occipital

With the exception of one small occipital fragment, six were cut-marked (N = 6, 83.3%) and of these, three had percussion marks and associated fracture notches. Cut-marks were mainly present on the squama of the occipital [cf. 37], [ 39]. In one case [GC87(190) + GC87(162)], para-sagittal cut-marks were concentrated on the inferior nuchal line. Slicing cut-marks occurred below the nuchal line on four occipital bones (Figure 2D, e). The location of these marks is indicative of cutting of neck muscles (*semispinalis capitis*, *rectus capitis posterior minus* and *major*, *obliquus superior*, *trapezius*) during detachment of the head. On three occipital fragments short cut-marks were located around the foramen magnum and in one case on the basilar portion of the occipital. The distribution of these cuts, close to the insertion of the *longus capitis* and *rectus capitis anterior* muscles, provide additional evidence for the detachment of the head.

There is no evidence of intentional breakage at the base of the skull in proximity to the foramen magnum. However, percussion marks and associated fractures were located in the region of the superior [in GC86(55)A and in GC87(190) + GC87(162)] and inferior nuchal crests in GC2 (Figure 2D, f). These are associated with flaking and chipping of the edge (Figure 4).

### Facial bones

**Figure 5:**
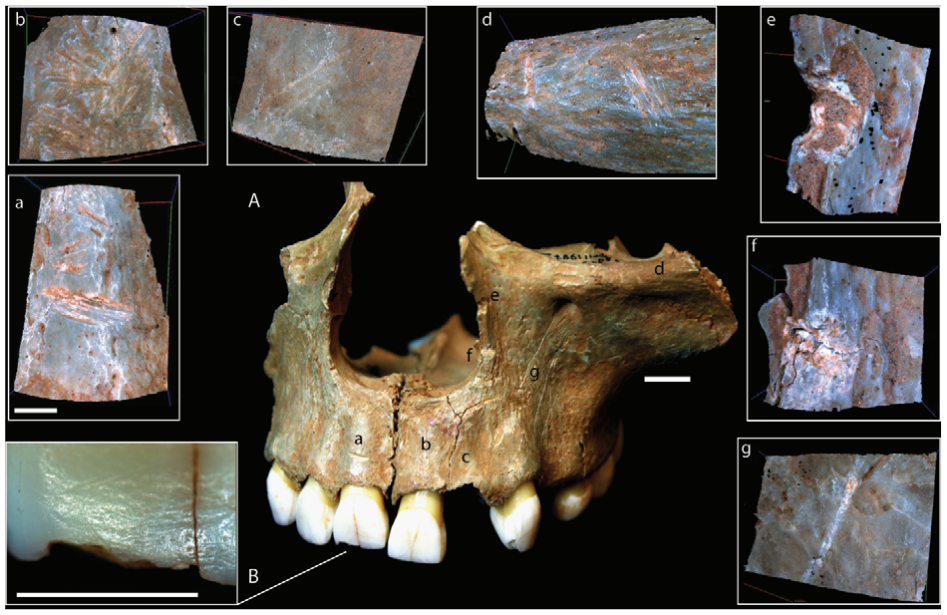
Cut-marks and percussion marks on facial bones from Gough's Cave. (A) Cut-marks and percussion marks on facial bones from Gough's Cave [GC 87(25), GC 87(29) and GC 87(87)] (scale  =  10 mm). (a–g), Alicona 3D images of human modifications (scale  =  1 mm). (B), photograph of percussion marks on the inferior labial border of the right central incisor (scale  =  5 mm).

Three zygomatics, two nasal bones and five hemi-maxillae were identified in the collection. Facial bones were generally intensively cut-marked (Figure 5A). The only unmarked pieces are two small fragments of nasal bone. In contrast, all zygomatic fragments were cut-marked and two have percussion marks.

On all zygomatic bones, perpendicular slicing cut-marks were located on the inferior border of the orbits and on the maxillary process [cf. 38], [ 41], [ 45]. These indicate severing of the *orbicularis oculi* muscle and extraction of the eye from the orbit socket (Figure 5d). In two cases [GC87(226) and GC87(230)A], clusters of perpendicular cut-marks were located on the inferior border of the zygomatic bone, at the origin of the *masseter* muscle [cf. 42], [ 46]. In the case of GC87(230)A, these slicing cut-marks were associated with percussion marks on the anterior portion of the inferior border. Percussion marks were also observed along the temporal border at its junction with the temporal process of the zygomatic bone.

On the hemi-maxillae, parallel slicing cut-marks were present on the antero-inferior root of the zygomatic process, presumably also associated with the removal of the *masseter* muscle [cf. 37], [ 44], [ 46]–[47]. In the two almost complete maxillae - GC87(139) and GC87(87), sub-horizontal cut-marks were present along the alveolar process above the canines and incisors [cf. 38]. One particularly deep sub-horizontal slicing cut-mark was present above the right central incisor. This was matched by a series of oblique cut-marks on the alveolar area, corresponding to the left central and lateral incisor (Figure 5a–c). These cut-marks suggest cutting of the lips. In the case of two maxillae, the front teeth [central and lateral right incisors of GC 87(25) (Figure 5B); central and lateral right incisors, lateral left incisor and left canine of GC 87(139)] also showed scratches and percussion fractures on the inferior border of their labial surfaces.

Marks on both sides of the border of the nasal fossa in GC87(87) (Figure 5e, f) have characteristics reminiscent of chopping or percussion damage [cf. 38]. Long oblique cut-marks on both canine fossae in GC87(87) (Figure 5g) indicate cutting of the maxillary attachments of the *buccinator* muscle to extract the cheek [26].

In two cases, cut-marks were present on the palatine process of the maxilla along the mid-line and in one case the cuts extend onto the palatine bones. These were produced during cutting of the *palatopharyngeus* muscle.

### Mandible

Three almost complete mandibles and a fragment of a left mandibular ramus were analysed. The body of each mandible was cut-marked on both the buccal and the lingual surfaces. On the buccal surface, cut-marks were concentrated along the oblique lines, and indicate cutting of the *buccinator* muscle. These marks support the interpretation of removal of the cheeks based on evidence from cut-marks on the facial bones. Cut-marks were also present in the area around the gonial angle and along the inferior border of the body of the mandibles [cf. 48]–[49]. These could have been produced during detachment of the head. Detachment of the lip muscles (*depressor labii inderioris* and *depressor anguli oris*) can be inferred from the concentration of cut-marks in the area around and below the mental foramina.

**Figure 6:**
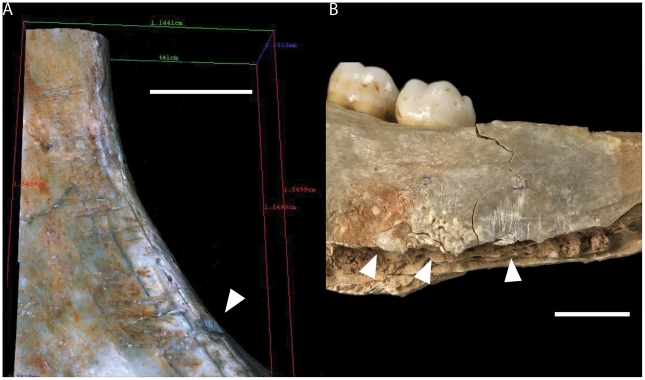
Cut-marks and percussion marks on mandibles from Gough's Cave. (A) Alicona 3D image of cut-marks and percussion mark (white arrow) along the anterior border of the mandible ramus of GC87(253), scale  =  5 mm. (B) Lunate scars (white arrows) on the lingual surface of the body of the mandible of CG(49); scale  =  10 mm.

Four of the five rami of the mandible have cut-marks along the anterior border, probably inflicted as a result of *masseter* muscle removal and/or detachment of the *temporalis* muscle from the mandible [cf. 19], [ 37], [ 50]. In two cases these were associated with discrete percussion marks (Figure 6A).

On the lingual surface, cut-marks were concentrated along the mylohyoid lines and around the spine of the mandible [cf. 16], [ 19]. Cut-marks in these locations indicate cutting of the tongue and hyoid muscles (*mylohyoid*, *genioglossus*, and *geniohyoid* muscles) and consequent removal of the tongue. In one example (Gough's Cave 6 -1.1/3), cut-marks were observed at the insertion of the *medial pterygoid* muscle, further suggesting detachment of the mandible from the skull.

Percussions marks were present on two of the mandibular bodies. Mandible GC 87(49) exhibits clear impact damage that removed the posterior portion of the inferior border on both sides. Modifications include lunate scars with pits/hammerstone damage on the lingual surface and anvil scratches on the buccal surface. The cracks and fractures suggest that the blows were inflicted on the lingual surface (Figure 6B). Evidence of impact damage is less obvious in GC6 where percussion marks are present only on the inferior border of the left ramus, but not on its counterpart. In this case, however, the blows appear to have been inflicted on the buccal surface. GC 86 (unnumbered) is a fragment of the anterior border of the left mandibular ramus and associated coronoid process. There is a possible impact pit on the oblique line and a series of deep abrasions were also present. The latter may relate to contact between the mandible and an anvil during processing.

### Processing of the head

The distribution of the cut-marks and percussion damage on the Gough's Cave cranial sample indicates the skilled post-mortem processing of the head. This included careful removal of soft tissues and controlled percussion. Cut-marks on the areas of insertion of neck muscles and the presence of cut-marks in proximity to the foramen magnum indicate that the head was detached from the body at the base of the skull. This is confirmed by the distribution of cut-marks on the axis and atlas vertebrae, which indicate dismemberment of the neck and head [26]. It is likely that this took place shortly after death, before desiccation of the soft tissues or decomposition and natural disarticulation had occurred [51]. The presence of cut-marks on the areas of insertions of the *medial pterygoid* muscle (both on the sphenoid and the mandible) indicate subsequent detachment of the mandible from the skull. In the case of the two maxillae, the front teeth showed post-mortem scratches and percussion fractures on the inferior border of their labial surfaces. Although non-masticatory scratches on front teeth are well documented [e.g. 52]–[56], descriptions of percussion modifications are rare in the literature [37], making it difficult to interpret their significance. Because of the taphonomic and sedimentological characteristics of the site [31], it is very unlikely that these modifications were naturally produced by sediment pressure or trampling. Neither can these marks be attributed to post-excavation cleaning or instrument damage. If associated with the processing of the head, it is possible that scratches and breakages were induced by a lever inserted between the occlusal plane of the front teeth, in order to disjoint and separate upper and lower jaws. The distribution of cut-marks on the temporal, sphenoid, parietal and zygomatic bones indicate removal of the major muscles of the skull (*masseter* and *temporalis*). The location of cut-marks in discrete areas such as the lingual surface of the mandible, the alveolar process of the maxilla, the root of the zygomatic process on the temporal bone and along the fronto-nasal suture, indicates that the tongue, lips, ears, and nose were also removed. Cut-marks around and inside the eye sockets and on the malar fossae of the maxilla suggest extraction of the eyes and cheeks. Finally, the high incidence of oblique para-sagittal cut-marks on the vault, in areas far from the attachment of muscles, on the squama of the frontal and on the parietals on both sides of the sagittal suture, suggests scalp removal. All these modifications are indicative of meticulous removal of the soft tissues covering the skull. The final stage in the sequence of alterations involved controlled percussion resulting in a systematic pattern of removal of the facial bones and the cranial base with minimum breakage of the vault. The distribution of impact damage and flaking is indicative of carefully controlled chipping of the broken edges in order to make them more regular (Figure 4).

## Discussion

**Figure 7:**
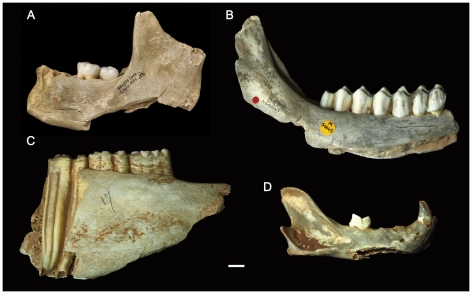
Modification of human and non-human mandibles from Gough's Cave. Human and non-human mandibles showing similar breakage patterns indicative of marrow extraction. (A) human; (B) red deer (*Cervus elaphus*); (C) wild horse (*Equus ferus*); (D) lynx (*Lynx lynx*). Scale 10 mm.

While so far unique in Britain, the post-mortem cranial modifications observed at Gough's Cave fit well within a Magdalenian context (Figure 1). Cut-marked and broken human bones are known from other Magdalenian European sites [19], [57]–[60] particularly in the Dordogne area in France and the Rhine Valley in Germany. The modifications of human bones at these sites have been more often interpreted as indicative of secondary burials. At Isturitz, for example, the evidence of a higher frequency of human cranial remains compared to those of other animals was used to refute the hypothesis of cannibalism [20] and suggest a ritual use of the human skulls. Similarly, at Gough's Cave, human skulls are well-represented in the collection, while non-human skulls are practically absent. This pattern could indicate differences in the way non-human and human bodies were treated. However, human and non-human remains were found discarded in the same archaeological context and exhibit similar treatment. For example, a comparison of modifications on human and non-human mandibles indicates that they were treated in a similar way: the mandibles were severed from the head, and carefully defleshed and broken, mainly along the angle of the mandible (Figure 7). These processes are consistent with bone marrow extraction, and suggest a common butchery practice aimed at extracting edible tissues from both human and non-human carcasses [35], [37], [50].

An unusual characteristic compared to other sites for which cannibalism has been suggested [5], [35], [37], [50] is the high frequency of cut-marks on the Gough's Cave human bones. Here, cut-marks are often clustered, and rarely occur as isolated incisions. In other samples where high frequencies of cut-marks on human bones have been observed, post-mortem damage to the facial region has been interpreted as a mutilation practice [61]. In the case of Gough's Cave, however, there was no obvious sign of inflicted trauma either on cranial or post-cranial elements, which makes the hypothesis of mutilation of bodies unlikely. High frequencies of cut-marks could also be an indication of ‘stiffness’ of the carcass due to *rigor mortis* or freezing [62]. In the sample from Gough's Cave, scratches and percussion fractures on the labial surfaces of the front teeth could be an indication that the cadavers were processed when they were in a state of rigidity [63]–[64]. Another unusual characteristic of the Gough's Cave sample is the completeness of the cranial vault and the pattern of impact damage which contrast with sites where skulls have been broken to expose the endocranial contents for consumption. At sites where nutritional cannibalism has been documented, the cranial bones are invariably highly fragmented with impact damage often located on the top of the cranial vault [35], [37].

**Figure 8:**
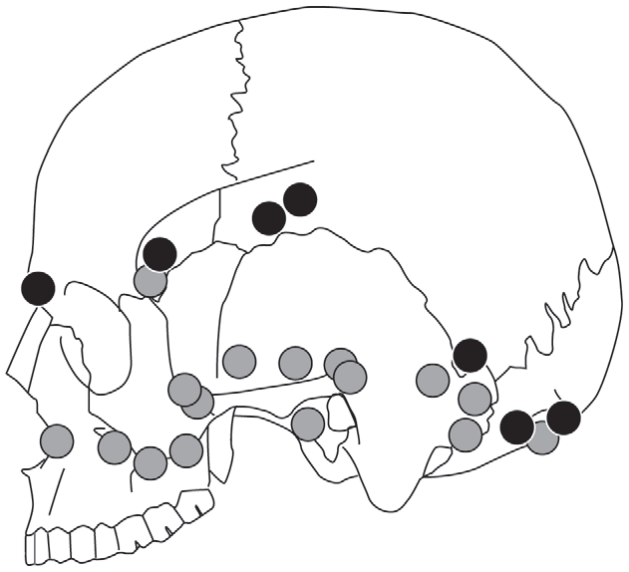
Distribution of percussion marks on cranial bones from Gough's Cave. Distribution of percussion marks on the vault (black dots) and on smaller cranial fragments (grey dots).

A more likely explanation for the high frequency of cut-marks and completeness of the vaults is that the skulls were scrupulously prepared or ‘cleaned’ using flint tools as an initial stage in the manufacture of skull-cups. Scalping and defleshing was followed by removal of the basicranium and facial regions and shaping of the vault using a hammerstone and anvil. Initially, the facial bones were detached from the neuro-cranium. This is indicated by percussion damage on the fronto-nasal suture, in the region of the fronto-sphenoid suture, on the root of the zygomatic process, and around the anterior portion of the squamosal suture. Percussions pits on the temporal bones appeared to follow a plane joining the middle point of the spheno-temporal suture, the root of the zygomatic process and the parietal notch. On the occipital bone, percussion pits created sub-horizontal fractured margins that contour the nuchal crests. Percussion marks on the neuro-cranium were inflicted in discrete clusters and their location approximates a sub-horizontal plane joining the *nasion* to the *inion* (Figure 8). These percussions resulted in a large number of small pieces from the cranial base, none of which can be refitted to the more complete cranial vaults. The presence of these smaller fragments suggests processing of the skull was undertaken within the cave. The distribution of percussion features indicates controlled removal of the facial region and cranial base, with minimal breakage of the vault. Completely defleshed and skinned skulls were probably essential in order to perform such a meticulous breakage technique. A similar breakage pattern has been observed at the Neolithic site of Herxhein in Germany [14]–[16], where fracture edges have also been observed along the same sub-horizontal plane. In contrast to Gough's Cave, the Herxhein skulls exhibit few cut-marks, suggesting that the skulls were shaped after the soft tissues had decayed, possibly months or years after the death of the individual [14].

At Gough's Cave, the impact damage on the neuro-cranium is located on the ectocranial surface of the bones. In two cases the impacts were associated with anvil scars and flaking on the endocranial surface, suggesting that the skull was placed on an anvil before it was struck. The overall pattern of flaking, which includes irregular scalloped breaks on both GC87(190) + GC87(162) and GC3 [1.1/1], is indicative of carefully controlled chipping to straighten the rim of the skull-cups (Figure 4). Similar modifications have been described for the Magdalenian sites of Le Placard (Charente, France) and Isturitz (Pyrénées-Atlantiques, France). At Le Placard, numerous long cut-marks were also located along the parietal and frontal bones. The high frequency of cut-marks on the vault is comparable with that observed at Gough's Cave [19], [65]. At Le Placard, blows inflicted on the frontal, temporal, parietal and occipital bones produced almost complete calottes. In the case of skull B and skull C, regular ‘retouch’ produced an even border around the edge of the calottes [18]. Le Mort and Gambier [19], [65] concluded that most of the calottes were deliberately broken to make ‘*goblets’* or ‘*coupes crâniennes*’. Similarly at Isturitz, four calottes have been described as ‘*coupelles*’ [20]. These have also been intensively cut-marked, carefully broken in order to preserve most of the upper portion of the vault, and the broken borders made regular and in some cases polished. In some remarkable cases, the calottes were engraved with animal representations (20, Fig. 2, 3 pages 173–174). Le Mort [66] suggested that the modifications (preparation of skull-cups and engraving) were produced immediately after the bodies were actively defleshed.

### Conclusion

**Figure 9:**
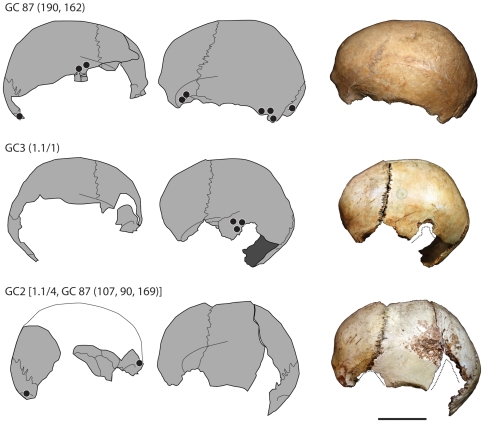
Skull-cups from Gough's Cave. Skull-cups from Gough's Cave showing the distribution of percussion marks (black dots) and post-deposition damage and recent breaks (dotted line). Scale 50 mm.

Three skull-cups have been identified amongst the Magdalenian human bones from Gough's Cave. The best preserved is CG87 (190) + GC87 (162), from the skull of an adult individual (possibly male). GC2, also the vault of an adult individual, has some post-depositional damage, including some recent breaks. The completeness of the cranial vault and the distribution of cut-marks and percussion marks suggest that the skull was modified in a similar way to CG87 (190) + GC87 (162). GC3, an almost complete vault of a child about 3 years old, also has received similar treatment, with the complete removal of all soft tissues and careful breakage of the skull (Figure 9). The striking similarity of the Magdalenian skull-cups at Gough's Cave, Le Placard and Isturitz to ethnographic examples (e.g. http://www.lightstalkers.org/images/show/137862) suggests they were also used as containers or drinking-cups.

The Magdalenian is associated with widespread evidence for the artificial modifications of human remains. This contrasts with earlier Upper Palaeolithic periods such as the Gravettian, where primary inhumation (sometimes with elaborate grave goods) was the common burial practice [67]. Earlier interpretations of Magdalenian cut-marked human bones have implicated ritual practices involving disarticulation, defleshing and excarnation, but the consumption of the human tissues has generally been dismissed. At only two other Magdalenian sites (Le Placard and Isturitz) has the production of skull-cups been described. Both assemblages have an over-representation of cranial elements, many of which are intensively cut-marked. This evidence has been interpreted as ritual mortuary practice intended to prepare skull-cups.

At Gough's Cave there is unambiguous evidence for the intentional controlled production of skull-cups, resembling those from the Le Placard and Isturitz as well as modern ethnographic examples [8]. The distribution of cut and percussion marks, however, suggests that this meticulous shaping of the cranial vault was preceded by the processing of the cadavers for consumption of body tissues (including bone marrow from the mandible), with a pattern of cuts and impact damage that is identical to that found on other large mammals from the cave [26]–[27].

The combination of cannibalism and skull-cup production at Gough's Cave is so far unique in the European Upper Paleolithic. Direct determinations on two of the vaults (∼14,700 cal BP) make these the oldest dated examples of skull-cups in the archaeological record.

## Materials and Methods

Gough's Cave is located in Cheddar Gorge in south-west England (Figure 1). Interest in the cave as an archaeological site dates from the 19^th^ century [28], [68]–[69]. Between 1987 and 1992 rescue excavations in the cave were undertaken by archaeologists and palaeoanthropologists from the Natural History Museum (London), and Nottingham University. During these campaigns two small undisturbed areas of sediment (area I and III) were excavated. The modified human remains described in this paper predominantly derive from these more recent excavations, but a number also come from earlier controlled and uncontrolled excavations in adjacent regions inside the cave entrance (see Table 1).

Human and faunal remains were initially analyzed using a hand lens and binocular microscope. An Alicona InfiniteFocus optical surface measurement system [43], [70] was used to produce detailed three-dimensional micromorphological models of cut-marks (vertical resolution 1 µm < z < 5.3 µm and a lateral resolution 1.75 µm×1.75 µm). Modifications of bone surfaces were recorded as slicing cut-marks [71] and percussion marks: percussion pits, lunate scars, anvil striae, anvil fractures and adhering flakes [36]–[37], [72].

## Supporting Information

Figure S1 Cut-marks and percussion marks on human skull-cups from Gough's Cave.
(EPS)

Figure S2 Cut-marks and percussion marks on human neurocranial bones from Gough's Cave.
(EPS)

Figure S3 Cut-marks and percussion marks on human facial bones from Gough's Cave.
(EPS)

Figure S4 Cut-marks and percussion marks on human mandibles from Gough's Cave.
(EPS)
